# Thoughts that breathe, and words that burn: poetic inquiry within health professions education

**DOI:** 10.1007/s40037-021-00682-9

**Published:** 2021-09-01

**Authors:** Megan E. L. Brown, Martina Kelly, Gabrielle M. Finn

**Affiliations:** 1 Health Professions Education Unit, Hull York Medical School, University of York, York, UK; 2 Medical Education Innovation and Research Centre, Imperial College London, London, UK; 3 Department of Family Medicine, Cumming School of Medicine, University of Calgary, Alberta, Canada; 4 School of Medical Sciences, Faculty of Biology, Medicine and Health, The University of Manchester, Manchester, UK

**Keywords:** Poetic inquiry, Qualitative research, Phenomenology, Health professions education, Medical education, Poetry

## Abstract

Qualitative inquiry is increasingly popular in health professions education, and there has been a move to solidify processes of analysis to demystify the practice and increase rigour. Whilst important, being bound too heavily by methodological processes potentially represses the imaginative creativity of qualitative expression and interpretation—traditional cornerstones of the approach. Rigid adherence to analytic steps risks leaving no time or space for moments of ‘wonder’ or emotional responses which facilitate rich engagement. Poetic inquiry, defined as research which uses poetry ‘as, in, [or] for inquiry’, offers ways to encourage creativity and deep engagement with qualitative data within health professions education. Poetic inquiry attends carefully to participant language, can deepen researcher reflexivity, may increase the emotive impact of research, and promotes an efficiency of qualitative expression through the use of ‘razor sharp’ language. This *A Qualitative Space* paper introduces the approach by outlining how it may be applied to inquiry within health professions education. Approaches to engaging with poetic inquiry are discussed and illustrated using examples from the field’s scholarship. Finally, recommendations for interested researchers on how to engage with poetic inquiry are made, including suggestions as to how to poetize existing qualitative research practices.

**Supplementary Information:**

The online version of this article (10.1007/s40037-021-00682-9) contains supplementary material, which is available to authorized users.

A Qualitative Space highlights research approaches that push readers and scholars deeper into qualitative methods and methodologies. Contributors to *A Qualitative Space *may: advance new ideas about qualitative methodologies, methods, and/or techniques; debate current and historical trends in qualitative research; craft and share nuanced reflections on how data collection methods should be revised or modified; reflect on the epistemological bases of qualitative research; or argue that some qualitative practices should end. Share your thoughts on Twitter using the hashtag: #aqualspace.

## Introduction

*“Poetry is thoughts that breathe, and words that burn”*

Thomas Gray

Poetry is a vehicle for human meaning making. It is part of everyday life which, if you look, you’ll find in advertisements, music, even sports cheers. It is not a modern fascination—indeed, poetry likely predates written text [1]. Before humans documented their experiences in a written format, poetry was an integral part of communities’ oral cultures, used to pass knowledge, history, and stories between generations [2]. Poems condense human experience, and when this experience speaks to you in some way, poetry feels meaningful and important [3]. Given the innate resonance of poetry to the human condition, poems feature within higher education, too. Within health professions education (HPE), poems have been most typically integrated into curricula within medical humanities modules as a way of centering patient narratives, engendering empathy amongst trainees, and facilitating reflective practice [4–6]. Several medical and medical humanities journals publish authors’ poems as arts contributions, demonstrating an appetite for poetry as a creative outlet in HPE. Yet, despite the pertinence of poetry to human experience, poetry as a form of *research* has not been a central topic of HPE conversation. In this article we introduce readers to poetic inquiry and its potential to expand qualitative research within HPE. We start by offering a broad definition of poetic inquiry, consider why it is both necessary and well suited to the field, and situate our exploration within phenomenology. We then progress to outline some approaches to engaging with poetic inquiry and illustrate these approaches using examples from HPE scholarship. Finally, we invite interested readers to consider ways in which they could engage with poetic inquiry, or to consider ways in which they could poetize their existing qualitative research practices.

## What is poetic inquiry?

Poetic inquiry is an arts-based research methodology which treats poetry as a ‘vital way to express and learn’ by incorporating original poetry into academic research [7, 8]. Though there is no consensus definition, ‘the key feature of poetic inquiry is the use of poetry *as, in*, [or] *for* inquiry’ [9], with poetic inquiry employed in diverse ways by researchers to collect data, collect field notes, and represent and reinterpret data [7]. The approach is gaining traction in many social science disciplines, including sociology, psychology, anthropology, and education [10]. Reasons for the use of poetic inquiry include preservation of participant voice [7], to explore the relationship between language and meaning [11, 12], to deepen researcher reflexivity [13], and to increase the emotive impact and accessibility of academic research [14] as poetic inquiry can help both researchers and audiences engage more deeply with participant accounts [15]. Additionally, through its attention to language, poetic inquiry is a rigorous approach—it allows ‘thoughts [to] breathe’—whilst also promoting an emotive efficiency of qualitative expression which helps ‘words [to] burn’. Yet, despite the benefits poetic inquiry can offer qualitative research, it has not received the same degree of focus or enthusiasm within HPE.

## Why do we need poetic inquiry?

Creative deviation from specific analytic qualitative processes is not the norm within HPE. Though approaches such as phenomenology, which seeks to represent the rich and complex nature of participants’ lived experiences [16], are becoming increasingly important within HPE, there has been a move to solidify processes of analysis to demystify the practice, and so increase rigor [17]. Whilst an important consideration, we believe the pendulum of data analysis is in danger of swinging too far—from openness to fixity—and qualitative research is at risk of stagnating. Several scholars have noted that qualitative inquiry often tends towards the descriptive, with deep thought regarding the connections between data, and the meaning of these connections wanting [17, 18]. Such deep thought is necessary for approaches which seek to represent the complexity of experiences, such as within interpretative phenomenology [19]. Others have argued that servile adherence to one’s data through a rigid set of analytic steps leaves no time or space for moments of ‘wonder’, surprise, or the emotional responses of the researcher that facilitate rich engagement and interpretation [20–22]. Being bound too heavily by methodological processes represses the imaginative creativity of qualitative expression and interpretation that has traditionally been a cornerstone of the approach [23, 24]. As we amass increasing levels of knowledge regarding topics such as professional identity formation within HPE, rich, creative qualitative exploration is necessary to advance scholarship in the field and expand understanding regarding the nuances of the process. New ways of scrutinizing old problems may also help further knowledge [25]. We propose that *poetry* through the medium of* poetic inquiry* may offer one way in which to restore creativity and deep engagement with qualitative data to qualitative inquiry within medical education.

## Poetic inquiry as part of phenomenological inquiry

As an approach, poetic inquiry is broad and diverse. Scholars have situated their engagement with poetic inquiry within a variety of different qualitative traditions such as phenomenology [26], participatory action research [27], ethnography [28], narrative inquiry [29] and performative inquiry [30]. We suggest that, within HPE research, poetic inquiry finds a natural home as part approach of phenomenology.

Indeed, poetry is *essential* to phenomenological thought [31]. For Heidegger, thinking is something which *only* occurs in the company of, or with poetry [32], Van Manen refers to phenomenology itself as a ‘poetizing project’ [26], whilst Bachelard used poetry to consider the meaning and impact of physical spaces [33]. As researchers immerse themselves in the construction of poetry from data, they open themselves to ‘what is said, or more accurately to what is unsaid’ [32], a necessary condition to unconcealing phenomena which, for interpretative phenomenologists like Heidegger, is synonymous with truth [34, 35]. As Galvin and Todres surmise, ‘poetic language emphasizes ‘wholeness’, in that, through rhythm, repetition, and imagery, a wholeness is pointed to that is more than what is there’ [36]. In this way, poetic inquiry facilitates an openness to ‘the otherness of language’ [32], and acts as a way of thinking about one’s data in a way that engenders moments of wonder, and stimulates deep, analytical thought. Whilst at the heart of phenomenology is a commitment to represent experience ‘well’, all too often this is taken by medical educationalists as a mandate to retell events as they are reported by participants, instead of utilizing phenomenological inquiry to open a *space of understanding*. Phenomenological accounts are not always factual—they can, and often do, draw upon fictional accounts to create ‘anecdotes of experience’, which convey meaning that is hard to communicate using a language of facts [37]. Creating poems as part of phenomenological inquiry can be seen as a way of creating these ‘anecdotes of experience’. For Van Manen, the key requirement of an ‘anecdote of experience’ is that the experiential account—in the case of poetic inquiry, the poem—is plausible in its truth-value [37]. Good phenomenological writing means that the reader recognizes the plausibility of the experience, even if they have never personally experienced that moment or kind of event. This is referred to by Van Manen as ‘the phenomenological nod’, where the reader of an account nods in agreement as they read about the lived experience of others [38].

Poetic inquiry may also help combat the oversimplification of findings within phenomenological research, which Sass comments is a key danger of phenomenological study [39]. Phenomenologists must constantly strive against reductionist portrayal of their findings—as Kelly et al. posit ‘phenomenology seeks to represent human experience in all its complexity, rather than seeking to reduce, parse or operationalize it’ [40]; this can cause issues when scholars are faced with journal-mandated word counts for their qualitative research. Portraying one’s data as poetry is an efficient way of displaying results within a qualitative paper’s results section, without (if done carefully, as we will discuss below) succumbing to reductionism. The necessity of ‘razor sharp’ language in short poems can powerfully capture the human experience in fewer words than with traditional qualitative quote display [41]. As Tse suggests, poetic inquiry ‘works with rather than against the complexities of experience, which researchers are always mining for understanding that is not easily extrapolated’[42]; simply put, poetic inquiry efficiently communicates a study’s findings whilst conserving their complexity. Poetic inquiry may, therefore, go some way to countering the temptation of reductionism in regard to phenomenological research.

Given the alignment of poetic inquiry to hermeneutic (interpretative [43]) phenomenological traditions, and its potential to counteract some of the issues phenomenological researchers may encounter, poetic inquiry has been used within phenomenological inquiry within other branches of the academe [13, 26]. Within HPE, poetic inquiry is similarly suited to use within a phenomenological approach.

## Types of poetic inquiry

One can engage in poetic inquiry in many ways. Van Luyn et al. draw distinctions between participant-voiced poetry, autobiographical poetry, and research poetry [44]. These approaches to poetic inquiry are summarized in Tab. 1. In sum, participant-voiced poetry (sometimes referred to as ‘vox participare [10]’, ‘found poetry’ [11] or ‘data poetry’[45]) is the practice of creating a poem ‘solely from primary sources’ such as interview transcripts or written reflections [46]. The researcher shapes a participant’s words, ‘re-present[ing]’ them in poetic form [47]. Autobiographical poetry (sometimes referred to as auto-ethnographic poetry [45], or ‘vox autobiographica [10]’) is what one might expect—the construction of autobiographical poems that explore the experience of the researcher, ‘in order to gain insight into a particular research process’ [44, 48]. Research poetry (or ‘vox theoria [10]’), is sometimes classed as a subtype of autobiographical poetry. However, Van Luyn et al. class research poems as a distinct entity, drawing attention to the fact that they are literature-voiced poems, written specifically in response to literature or theory in a field [44]. Whilst they are created by a researcher, they are ‘not a direct expression of the researcher’s personal experience’ [44].

**Table 1:** Types of poetic inquiry and their benefits

Type of poetic inquiry	Description	Proposed benefits of the approach	Examples of research employing approach
*Participant-voiced poetry*	Sometimes referred to as ‘vox participare’, ‘found poetry’, or ‘data poetry’. Created from research transcripts. Researchers shape the words of participants’, redisplaying their own words in poetic form to convey meaning. There can be significant variation in the degree of poetic licence taken by researchers. Some portray participant words unchanged, in chronological order as they appear in transcripts; others modify word order and punctuation more freely, blending participant and researcher voices. May involve one or multiple voices Includes participatory action-research variants, where researchers and participants co-construct poems. Increasingly popular is the approach digital poetic inquiry, where digital text is used to create found poems May be used as a method of data presentation, as an elicitation technique within interviews, or as a method of member-checking interpretations with participants	– Preservation of participant voice, especially useful for conveying the experiences of individuals who have been previously overlooked within academic discourse. In this way, this approach can challenge dominant ideologies within a field – Moves beyond simple description of events and experiences towards human struggles of being and becoming – Can challenge researchers’ biases and preconceived judgments of underserved populations – Insight afforded into complex psychosocial situations and processes can engender empathy – Emotive impact of poetic representation – Increased accessibility of findings to wider audiences	Brown CS. The use of poetry in qualitative post-hoc analysis. J Poet Ther. 2018;31:107–12
			MacNeil C. The prose and cons of poetic representation in evaluation reporting. Am J Eval. 2000, 21:359–67
			Poindexter CC. Research as poetry: A couple experiences HIV. Qual Inq. 2002;8:707–14
*Autobiographical poetry*	Sometimes referred to as auto-ethnographic poetry, or ‘vox autobiographica’. Poetry about the experience of the researcher to gain insight into the research process. Constructed from researcher field notes or journal entries	– Poetic inquiry is a method of embodied interpretation. In all its forms, writing helps researchers find out more about themselves and their topic of inquiry. Autobiographical poetry, in particular, can aid researcher reflexivity, and advance the development of new approaches or methods	Naidu T. Autoethnographic realisation of legitimacy of voice: A poetic trail of forming researcher identity. Qual Soc Res. 2014;15: 10.17169/fqs-15.1.1996
			Furman R. Using Poetry and Narrative as Qualitative Data: Exploring a Father’s Cancer Through Poetry. Fam Syst Health. 2004;22:162–70
			Rapport F, Hartill G. Crossing disciplines with ethnographic poetic representation. Creat Approach Res. 2012;5:11
*Research poetry*	Some have argued research poems are a form of autobiographical poetry. Many, however, consider this a separate type of poetic inquiry, which involves the creation of poetry from a field’s literature base or theory. It is sometimes termed ‘vox theoria’	– Can be used within critical poetic inquiry to cast new light on the dominant ideologies of a field and explore power – Can act as a form of review, synthesising bodies of literature or discourse surrounding a theory into a more accessible format	Lahman MK, Richard, VM. Appropriated poetry: Archival poetry in research. Qual Inquiry. 2014;20: 344–55
			Prendergast M. ‘Shaped like a question mark’: found poetry from Herbert Blau’s The Audience. Res Drama Educ. 2004;9:73–92
			Prendergast M. Found poetry as literature review: Research poems on audience and performance. Qual Inq. 2006;12:369–88

## How to ‘do’ poetic inquiry

Van Luyn et al.’s review suggests that participant-voiced poetry is the most common form of poetic inquiry [44]. There can be significant variation within this approach. Whilst we aim to cast some light on possible ways of engaging in participant-voiced poetic inquiry, the approaches we outline are by no means comprehensive or singular and are not intended to act as a prescriptive ‘how-to guide’. We outline two approaches to participant-voiced poetry: 1) Glesne’s method of poetic transcription and 2) Gilligan et al.’s listening guide, drawing on examples from HPE to illustrate their use.

### Glesne’s method of poetic transcription

We suggest Glesne’s popular approach to poetic transcription [49] may act as a springboard for HPE researchers interested in poetic inquiry, by making clear the process of abiding by clear inquiry principles. The first principle Glesne sets is that only the participant’s words may be used within her participant-voiced poems [49]. Additionally, the syntax (the grammatical structure of words and phrases within a sentence—in essence, the *ordering* of words) of a participant’s way of speaking should be persevered in the poems that are written. For example, though the phrases ‘the cat ran quickly’, ‘the cat quickly ran’, and ‘quickly, the cat ran’ technically convey the same meaning, their syntax differs, which emphasizes different words within the phrases and alters the voice of the sentence. Though Glesne allowed herself to ‘pull … phrases from anywhere in the transcript and juxtapose them’, they paid attention to the participant’s ‘speaking rhythm … [their] way of saying things’ and echoed this in their poems [49].

Practically speaking, Glesne’s process first involves coding and sorting, similarly to the start of many qualitative research analyses. Major themes are then generated to group clusters of codes. It is after this point that poetic writing starts—all data under one theme is re-read, and the researcher reflects on the meaning and connections within the theme. Participant words within these data are used to portray meanings and connections. As researchers begin to write, it may become apparent that themes require some re-ordering. As poems are crafted, connections between data may become apparent that previously were hidden. As such, codes may shift categories or require refinement to attend to these new connections. Finally, researchers edit the participant-voiced poems, ensuring they speak to the meaning and complexity of the data.

We offer an example from our own research to demonstrate how transcript data can become a poem. Drawing inspiration from Glesne’s principles of poetic transcription, we used only the participant’s own words, and words from within one transcript, to construct participant-voiced poems for newly qualified doctors who had recently crossed the threshold into clinical practice [50]. As Glesne recommends, we were attentive to participant syntax—during our re-readings we made detailed notes in the margins of each transcript concerning participant tone, emphasis, use of pauses, rhythm and syntax. We also reviewed each transcript for repeating words, phrases, and expressions. An example of our process is depicted within the Table S1 of the Electronic Supplementary Material, where one participant-voiced poem (‘Friends are everything’) is displayed alongside the section of original transcript from which it is drawn. Words and phrases utilized in the poem are underlined.

### Gilligan et al.’s ‘Listening Guide’ method

Gilligan et al. developed the ‘Listening Guide’ method in 2003 as a way of paying particular focus to how participants talk about themselves (using their first person ‘voices’)[51]. This method involves creation of a type of participant-voiced poetry, which helps researchers consider participant identities and subjectivities [52]. The method encourages researchers to pay close attention to participant use of the personal pronoun ‘I’ and recommends the creation of ‘I-poems’ in the second phase of the listening method [52]. The steps of the listening guide method are detailed in Tab. 2. Typically, I‑poems are utilized only in the data analysis phase of the listening guide method [52], though some authors have used them to display results [53]. For examples of I‑poems, we recommend interested authors review the following references [51–55].

**Table 2:** Steps of the listening guide. Complied using information from [51, 54, 55]

Steps of the listening guide	Description
*Step 1: Listening for the plot*	During step 1, the researcher listens for the plot—for what is happening in a narrative. Gilligan recommends considering who is present, who is missing, the major and minor themes, emotional moments, prominent imagery, the stories that are told, breaks in the narrative, and the researcher’s embodied response to the participants’ narrative
*Step 2: Constructing I poems*	During step 2, the researcher reviews and underlines each first-person singular “I” pronoun utilised by the participant in their transcript. Subsequent accompanying words should also be underlined—there is no set number of words that should be included alongside the “I” pronoun, but the accompanying words should provide context as to what is meant. An I poem is then constructed by compiling all I statements in the same order they appear in a participant’s transcript. Each I statement is placed on a separate line. Sometimes researchers take a degree of poetic licence, and incorporate additional words to like ‘me’, ‘my’ and ‘myself’ to expand their analysis. The purpose of this step is to draw attention to the ways in which a participant speaks about themselves
*Step 3: Listening for contrapuntal voices*	Step 3 involves listening for the participants’ contrapuntal voices, the different voices present within their narrative. This step considers not only the content of a narrative, but tensions within an individual, harmonies or dissonances, and what is unsaid, or may be silenced. Contrapuntal voices have a relationship to one another, and this should be a focus of interest to a researcher—how do the multiple voices in one participants’ narrative interact? Gilligan recommends that a minimum number of two voices be sought in each narrative
*Step 4: Composing an analysis*	Step 4 involves the synthesis of all previous steps, and researcher interpretations, to produce an analytical report

It is important to clarify that participant-voiced poetic inquiry is, by nature, an inductive approach to data analysis. If researchers wish to use educational theory to inform their analysis of participant-voiced poems, we suggest they do so in a way which aligns with what Varpio et al. term a ‘subjectivist inductive’ orientation to theory and research, where researchers work ‘from data up to abstract conceptualizations’ [56]. One subjectivist inductive approach to theory, which Varpio et al. term ‘theory-informing inductive data analysis’ may be of particular use to authors interested in applying theory to the process of poetic inquiry. In this approach, researchers move from data to theory, starting out with the intent to ‘understand or explain a … phenomenon’, and later using theory as ‘an interpretative tool’ to make sense of created participant-voiced poems [56]. In their study of the experience of women who identify as lesbian, gay, or queer, Lambert uses Butler’s theory of passionate attachments to analyze a poem created from participant interview text, employing theory in such a ‘theory-informing inductive data analysis’ approach [57].

Though we have outlined two specific approaches to poetic inquiry, we encourage researchers to read widely, and to adapt the method of poetic inquiry they select to suit their context and study, if appropriate. Transparency of one’s methodological approach is key, and adaptors must ensure they adequately detail their process, and justify the steps they have taken. There is an ethical domain to participant-voiced poetry, and it is important that researchers define and hold themselves to a set of principles throughout the process of inquiry [58].

## Poetizing HPE qualitative research

We envision that increasing participant-voiced poetry use within HPE research could add depth to research questions which concern how participants speak about, and perceive, themselves, others, and their experiences. Through inviting researchers to engage deeply with participant accounts, poetic inquiry within HPE is not just a tool, but a *way of being* that gives rise to the space for wonder, surprise, emotions and creative expression and interpretation.

Though the benefits of poetry may appeal, researchers may not wish, or have capacity to, dive head-first into the unknown waters of poetic inquiry. If this is the case, there are several ways in which one can engage in ‘methodological borrowing’ [59] to poetize more traditional qualitative research projects and develop as a poet gradually over time [60]. Reflexive and analytic journaling is common within qualitative research. Researchers could expand this practice to include *poetic* journaling, an approach where researchers begin to think poetically about their findings. No two poetic journals will look the same. Researchers may wish to read poetry, taking notes on impactful lines and phrases which make them think. Researchers may also begin to write poems about their data and position as a researcher, experimenting with participant phrases, their own thoughts, and poetic conventions like rhyme and form. Keeping a poetic journal may also help capture what the sociologist Mills terms ‘fringe thoughts’—ideas which are by-products of everyday life, and which we usually dismiss as ‘mental noise’ but can hold intellectual value relevant to data analysis and theorization, as they prompt us to think differently about our research [61]. Excerpts from poetic journals could also be included within research manuscripts to demonstrate the process of reflexive engagement researchers have undertaken throughout a project. Using Gilligan’s listening guide as part of one’s analysis, but not results representation, may help researchers begin to think poetically about their data, and develop their poetic ear, without fear of their poetic output being subject to wide scrutiny.

## Considerations of quality

There is debate within the poetic inquiry community as to what qualifies one to use poetic inquiry, with some concluding that, to engage in poetic inquiry, one should be a published or formally trained poet in their own right [58]. Respectfully, we disagree. Though proponents may claim their views are to protect the reputation of poetry, and ensure quality inquiry, such views position poetry as an elitist pursuit which prevents the engagement of novice, but keen, would-be poetic researchers. Given the benefits of poetic inquiry, we join with Lahman and Richard in advocating for ‘good enough’ research poetry [62]. ‘Good enough’ poetry is the space between the belief that ‘any person may write poetry’ (we recognize you need a grounding in poetic inquiry, and an appreciation of the approach), and ‘the scholarly belied that in-depth training is needed’ [62].

This debate may leave you feeling uneasy. As health professions educators, we enjoy practical guidance. If you are wondering how you will know whether your poems are ‘good enough’, we suggest you turn to Sullivan’s 2009 architectural dimensions of a poem [63] for reassurance, which Pate has synthesized into a quality checklist [26] (summarized in Table S2 of the Electronic Supplementary Material). As previously, these are not the only markers of poetic quality, nor do we believe they should be used prescriptively. Sullivan’s dimensions interplay and exist as a complex web or nexus that influence the impact and resonance of a poem. We advise that where researchers wish to use this checklist, they frame it as a broad guide or reflexive tool to consider the steps taken within their own research projects carefully and critically. Additionally, as researchers open up the possibilities of the method, it may be beneficial to learn more about typical poetic conventions such as form, meter and rhyme to advance your craft—but this is no means a prerequisite, at least in our eyes, to the ability to produce ‘good enough’ participant-voiced poetry.

## Concluding thoughts

Though qualitative research is increasingly valued, barriers still exist to the progression of qualitative research within HPE. Discussions of the quality of qualitative research have centered methodological rigor, an important concern, but one that has dominated conversation. The threat to qualitative creativity has been much less frequently discussed within HPE. Poetic inquiry has not been a central topic of conversation yet holds the potential to encourage researchers to think deeply and creatively about their findings. Poems emphasize language and may cast new light on areas of HPE where thinking has become narrowed. Poetic inquiry can foster increased researcher attention to reflexivity in a creative and contemplative way. Crucially, poetic inquiry can preserve participant voice within research reports, and may offer one way to represent experience more faithfully. Poetic inquiry has much to offer HPE, and we encourage researchers to take up the poetic mantle to diversify research within the field and cultivate creativity. For, to return to the words of the poet Thomas Grey, who would not wish their thoughts to breathe, and words to burn?

## Supplementary Information

Supplementary table 1: Construction of a participant-voiced poem in our own research using Glesne’s method of poetic transcription. Supplementary table 2: Sullivan’s architectural dimensions of a poem, as synthesised into a quality checklist by Pate
