# Supplementary material for: Thoughts that breathe, and words that burn: poetic inquiry within health professions education
Source: Perspect Med Educ. 2021 Sep 1;10(5):257–64. doi: 10.1007/s40037-021-00682-9 (PMC8505582; doi:10.1007/s40037-021-00682-9)
Supplement: Supplementary file 1 — Supplementary table 1: Construction of a participant-voiced poem in our own research using Glesne’s method of poetic transcription. Supplementary table 2: Sullivan’s architectural dimensions of a poem, as synthesised into a quality checklist by Pate [file 40037_2021_682_MOESM1_ESM.docx]

Supplementary material:

Supplementary table 1: Construction of a participant-voiced poem in our own research using Glesne’s method of poetic transcription:

| **Excerpt from in-depth interview transcript**  *And, I wondered whether there had been any moments in those first few months where you felt like you particularly belonged?*  The real stand out moments for me were when I was with the other F1s - we actually fairly quickly became friends. And I think came to mean a lot to each other, you know, I think most people would say that friends are really important and that to me hit home during F1. Like, when we spent time together, it was just like we were doing everything together even though we worked in different places, you know?  *Hmm, yes.*  I am honestly just so grateful that they were a good group of people and that our personalities just seemed to gel in a way that we ended up being quite close-knit.  *Did you get to see them much, you said you all worked in different places…*  It was quite variable, obviously we had the lunchtime teaching once a week but there wasn’t much time for catching up because we were just focused on eating and trying to avoid eye contact with whichever consultant was giving the session [Laughter]. But we did have catch ups outside work, those of us who lived close. Normally at the pub [laughter], it sounds bad.  *No, no it doesn’t. What sort of things would you catch up about?*  Mainly just, well, the extremes normally- the very good - very good and very bad. And it was, yeah, to use a medical term a bit of a debrief really, or a sounding board. You know, when things were going well, on good days we’d kind of be pleased for each other, for our friends, sort of cheer them on. And then when they were having bad days, when we were having bad days, we’d just sit sometimes quite quietly and be miserable together [Laughter].  *Oh no…*  No, it wasn’t as bad as I’m making it sound. We’d talk and try to think things through together, instead of just sitting at home alone feeling bad.  *Ah ok, so it sounds quite supportive…*  You know, thinking about it now it really was. I was quite hesitant, if that’s the right word, at first with them, like I was starting a new school, and I didn’t always fit in at school, but with them I did actually- which was nice. There was a nice sort of feeling that, no matter where you were from, or which uni you’d attended, or who you were before starting at the hospital, now you’re an F1, we’re all in the same boat basically, and we could just be friends, we instantly had that in common, even though we might not have been friends in school.  *And why do you think that is, how do you think it equalises things?*  It’s from necessity [Laughter]. F1 is so hard, the hardest, and you need to support each other because there is strength in numbers, and if something goes wrong you can not only debrief but also sort of- defend each other. Especially when it comes to things like… there was a harsh word we had off the programme director about it being said that some F1s weren’t seen to be doing enough at weekends because they hadn’t been seen on the wards. And we really had to clump together and be like “no, that’s because we’re spread so thinly… they don’t see us because we’re trying to be everywhere”, you know?  *So that support you had from the other F1s could be a sort of protection too?*  Yes, that’s a good way of putting it.  *So, a space to debrief and a defence against any sort of unjustified criticisms, do you think there were any other benefits to that network you developed?*  Another part of it, and it might come into that debriefing bit, is that it’s just nice to have something stable, especially when work feels so all over the place. Like we had in-jokes and we built up like- had experiences together and things, it was like we had our own little club, and that was actually quite nice when everything around you is probably different today than it was yesterday.  *Sure, so sort of that, continuity piece that was missing.*  Continuity, yes, but also humanity, I think. There are just so many times in F1 where you’re sort of referred to like that, no one uses your name or cares. Whereas in the pub as a group, we could sort of sit and with our backs to all that stress on the wards, we could finally just be us, just be normal people with names and regular lives. It feels like you’re a little cog sometimes and it was nice to just remind ourselves, more than anything, that we are people, we have lives that matter too.  *Gosh, yes, absolutely. It sounds like friendships played a really big part in getting you through those initial stages.*  Oh, definitely. I am such a firm believer that friends are so important, they have a bearing on everything that you do if you have a close enough connection, and I missed out on a lot of that in school, it was better in uni but still- I just felt so relieved when I met them all and realised that we’d get on, you know, it was like “Thank God”, “Thank God I found some friends”. | **Poem: “Friends are everything”**  Friends are everything  They were a good group  Close-knit  Good days we’d cheer  Bad days we’d sit  Think together instead of alone  I didn’t always fit in but with them I did  No matter who you were before  Now you’re an F1*  And there is strength in numbers  It was like our own little club  With our backs to the wards  We could just be us  Remind ourselves that we are people too  Friends are everything  Thank God I found some friends  **F1= Foundation year one doctor, first postgraduate year in the UK.* |
| --- | --- |

Supplementary table 2: Sullivan’s architectural dimensions of a poem, as synthesised into a quality checklist by Pate:

| **Architectural dimension** | **Description** |
| --- | --- |
| Concreteness | Poems should be concrete in that they bring images to life by providing the reader with a sensory or embodied experience. |
| Voice | Voice is a primary lyric quality, it is personal, authentic, and resonant with emotion and experience. Even if it doesn’t use concrete images to invoke embodied experiences in its reader, strong voices have a concreteness themselves. Interview data already represent an individuals’ voice. Quality participant-voiced poetry recognises this voice and reconstructs it in a poetic form. Syntax, rhythm, pauses and tone are all important considerations when translating the voice of an individual into a poem. |
| Emotion | Poems should convey emotions, evoking felt experiences. |
| Ambiguity | Poems should reveal ambiguity by exhibiting and revelling in paradoxes, multiple dimensions, complexity and contrapuntal layering. |
| Associative logic | Poems should be constructed to reveal the web-like relationships, connections and coherence of a particular phenomenon which defies linear texts and representations. This is a special sort of logic inherent to poetry, which requires us to redefine how we view and conceptualise coherence. |
| Tensions | Poems should unearth underlying tensions and conflict and bring these to the surface in their construction. There may be internal turmoil, tensions between what is intended and what is accomplished, tensions between multiple or single voices, or between individuals and establishments. |
